# “UPRegulation” of CD47 by the endoplasmic reticulum stress pathway controls anti-tumor immune responses

**DOI:** 10.1186/s40364-017-0105-8

**Published:** 2017-08-14

**Authors:** Katherine L. Cook, David R. Soto-Pantoja

**Affiliations:** 1 Department of Surgery, Wake Forest School of Medicine, Winston-Salem, NC 27157 USA; 2 Department of Cancer Biology, Wake Forest School of Medicine, Winston-Salem, NC 27157 USA; 3 Department of Radiation Oncology, Wake Forest School of Medicine, Winston-Salem, NC 27157 USA; 4 Wake Forest Baptist Comprehensive Cancer Center, Wake Forest School of Medicine, Winston-Salem, NC 27157 USA

**Keywords:** Unfolded protein response, CD47, Thrombospondin-1, GRP78, Tamoxifen, Endocrine therapy resistance, Innate anti-tumor immunity, Immunometabolism

## Abstract

We recently demonstrated that targeting the unfolded protein response (UPR) protein GRP78 down-regulates CD47 expression, resulting in increased tumor macrophage infiltration and inhibited resistance to anti-estrogen therapy. We now show new data indicating that anti-estrogen therapy regulates CD47 expression and implicates its ligand, thrombospondin-1, in regulation of tumor macrophage infiltration. Moreover, GRP78 and CD47 co-expression is associated with poor prognosis in breast cancer patients, suggesting the existence of crosstalk between UPR and immunity that regulates therapeutic responses in breast cancer.

## Main text

The unfolded protein response (UPR) is a highly conserved cellular response pathway in the endoplasmic reticulum. The UPR is aimed at preservation of correct protein folding and protein load during stress, and thus controlling both cell survival and death. We previously demonstrated that glucose regulated protein-78 (GRP78) mediates resistance to anti-estrogen therapy in estrogen receptor-positive breast cancer [1, 2]. A study published in *Cancer Research* by Cook et al. showed that GRP78 controls fatty acid metabolism by regulating mitochondrial lipid transport and sterol regulatory element binding protein-1 (SREBP1) transcription. GRP78 inhibition, alone or in combination with tamoxifen (an estrogen receptor-α targeting treatment), was accompanied by accumulation of cellular linoleate, linolenate, dihomo-linoleate, dihomo-linolenate, and arachidonate polyunsaturated fatty intermediates [3]. Inhibition of GRP78 or administration of linoleic acid sensitized breast tumors to anti-estrogen therapy [3]. Interestingly, these treatments also inhibited CD47 expression. CD47 is a widely expressed cell surface receptor that inhibits phagocytic signaling through engagement with its counter-receptor SIRPα on macrophages. CD47 also controls physiologic activities through its interaction with thrombospondin-1 [4]. Decreased CD47 expression in tumors after GRP78 targeting or administration of linoleic acid was associated with increased macrophage infiltration. Therefore, these results show a novel paradigm in which CD47 expression and function may be regulated by the UPR pathway and changes in lipid metabolism. This is the first report demonstrating that CD47 expression is regulated by UPR stress signaling.

These results have implications for findings by our group and others in models of stress and carcinogenesis. Cook et al. observed that increased macrophage tumor infiltration mediated by GRP78 blockade was associated with reduced CD47 expression. We previously made a similar observation in our in vivo melanoma and squamous lung tumor studies, in which direct targeting of CD47 using anti-sense morpholinos increased macrophage recruitment in tumors [5]. The same SIRPα/CD47 interaction that regulates macrophage phagocytic activity has been implicated in regulation of macrophage migration; however, the mechanisms remain largely unknown [6]. Studies assessing the intraepithelial and stromal macrophage population in over 200 primary colorectal tumors showed elevated tumoral CD68+ cell infiltration was associated with increased long-term survival and reduced lymph node metastasis [7]. Moreover, loss of CD47 was associated with increased CD68+ and CD163+ macrophage infiltration, which correlated with reduced tumor grade and lymph node metastasis [7]. Taken together, these data suggest that CD47 signaling in the tumor not only regulates phagocytic activity, but also mediates tumoral infiltration of macrophages.

Our recently published data implicate GRP78-mediated CD47 regulation as a possible molecular driver to promote anti-tumor macrophage recruitment [3]. Moreover, we showed that CD47 was highly expressed in endocrine therapy-resistant tumors, suggesting a new role for CD47 in mediating anti-estrogen resistance [3]. Tumor re-sensitization to anti-estrogen therapy mediated by GRP78 targeting was correlated with increased levels of calreticulin (CALR) and high molecular group box 1 (HMGB1) protein, known pro-immunologic cell death and phagocytic signals.

Previous studies demonstrated that the anti-phagocytic signal elicited by CD47 expression may be counterbalanced by co-expression of CALR [8]. Increased phagocytosis of tumor cells mediated by targeting CD47 was inhibited by CALR blockade and its interaction with low density lipoprotein receptor-related protein-1 (LRP1). We also demonstrated that the reciprocal regulation of CD47 and CALR was influenced by GRP78 [3]. These data provide evidence that activation of UPR and changes in lipid metabolism may directly modulate immune surveillance to inhibit anti-tumor immune responses. Because the highly proliferative nature of cancer cells requires synthesis of large amounts of nascent proteins, tumors often have elevated GRP78 and UPR signaling components [9]. The UPR-mediated upregulation of CD47 expression then may result in tumor expansion and insensitivity to anti-cancer therapies.

To further examine the relationship between UPR signaling and CD47, we used the KM-plot data base (kmplot.com) and mined the breast cancer dataset to assess whether co-expression of GRP78 and CD47 impact the relapse-free survival (RFS) of breast cancer patients [10]. The dataset, which contains Affymetrix arrays from 5000 patient samples, was mined using the JetSet best probe set; this scores each probe for specificity, splice isoform coverage, and robustness against transcript degradation, allowing measurement of gene expression levels [11]. This method uses the median gene expression as a cutoff for dividing samples into high- and low-expression groups [10]. The use of median for splitting minimizes the influence of outliers that distort the results when using the mean [11]. Also, use of the median as a cutoff enables high and low-expression groups of similar size, allowing the graphing of reliable Kaplan–Meier plots [10].

**Fig. 1:**
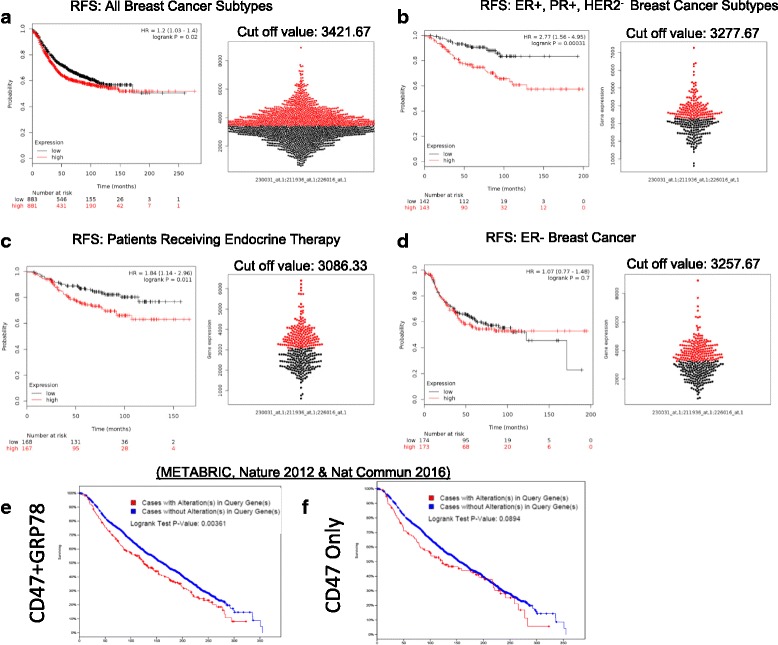
Co-expression of GRP78 and CD47 is associated with poor prognosis in breast cancer. Kaplan-Meier curves indicating relapse-free survival (RFS) of breast cancer patients were obtained by using KM plotter (2017 version for breast cancer) [10]. Effects of co-expression of GRP78 and CD47 on RFS are shown for (**a**) breast cancer patients regardless of hormone receptor and HER2- tumor status; (**b**) ER+, PR+, and HER2-negative breast cancer; (**c**) ER+ patients with ER+ tumors who endocrine targeted therapies; and (**d**) patients with ER- tumors. **e**, **f** Data mining of breast cancer cases using the METABRIC database (www.cBIOportal.org)

We found a statistically significant decrease (*p* = 0.02) in survival in all subtypes of breast cancer in over 1700 patients when both GRP78 and CD47 were co-expressed (Fig. 1a). A more robust effect on RFS was observed when GRP78 and CD47 were co-expressed in patients with estrogen receptor-α-positive (ER+), progesterone receptor-positive (PR+), and human epidermal growth factor receptor-2-negative (HER2-) tumors (Fig. 1b). In breast cancer patients who received endocrine targeting therapy, co-expression of CD47 and GRP78 was associated with reduced RFS (Fig. 1c). While over-expression of CD47 alone was previously found to be associated with reduced survival in ER- patients [12], GRP78 and CD47 co-expression was not associated with RFS in the ER- cohort (Fig. 1d), suggesting that co-expression of these two proteins may play a role in estrogen-mediated signaling to promote development of ER+ breast cancer.

We corroborated these results using the cBioportal database (http://www.cbioportal.org) and mining the Molecular Taxonomy of Breast Cancer International Consortium (METABRIC) [13, 14]. Results also showed an association between co-expression of CD47 and GRP78 mRNA (z-score threshold ±2.0 fold mRNA) and reduced overall survival (OS) in breast cancer patients (Fig. 1e). Expression of CD47 alone did not correlate with OS (Fig. 1f). These data strengthen the evidence behind our earlier observation that GRP78 may cross-talk with CD47 signaling to promote tumor growth [3]. Our KM plot data suggest that this interaction may be influenced by ER status or estrogen signaling, and that co-expression of these two receptors may mediate endocrine therapy resistance in patients with ER+ breast cancer.

**Fig. 2:**
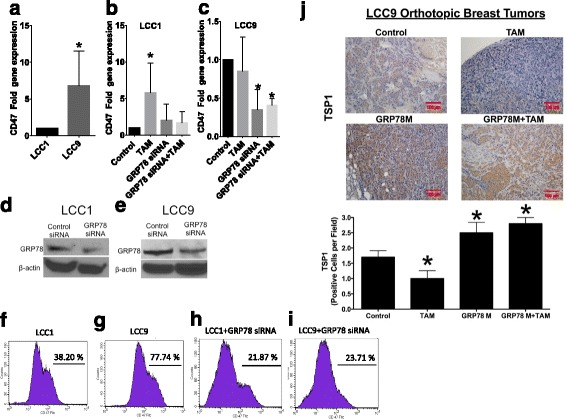
CD47 gene expression is associated with increased resistance to anti-estrogen therapy. **a** CD47 gene expression in LCC1 (tamoxifen-sensitive) and LCC9 (tamoxifen- resistant) breast cancer cells (*n* = 4 experiments, *p* < 0.03). CD47 expression in LCC1 (**b**) and LCC9 (**c**) cell lines transfected with control or GRP78 siRNA and treated with 100 nM tamoxifen for 72 h (*n* = 4 experiments, *p < 0.01). **d**, **e** Expression of GRP78 protein in LCC1 and LCC9 measured by Western blot hybridization. **f**-**i** Flow cytometry analysis of CD47 expression on the cell surface. **j** Immunoreactivity of TSP1 in LCC9 tumors harvested from mice treated with saline (control), 400 ppm tamoxifen citrate in chow (TAM), GRP78 anti-sense morpholino (GRP78M), or TAM plus GRP78M (*n* = 6/group, *p < 0.05)

Our new data generated in LCC1 (tamoxifen-sensitive breast cancer cells) and LCC9 (tamoxifen-resistant breast cancer cells) [15, 16] indicate that CD47 expression is increased in LCC9 cells, showing for the first time that CD47 expression correlates with resistance to anti-estrogen therapy (Fig. 2a). Tamoxifen-resistant LCC9 cells also have elevated levels of GRP78 compared to tamoxifen-responsive parental LCC1 cells [2], further supporting the concept of co-regulatory actions between CD47 and GRP78. Moreover, 100 nM tamoxifen stimulated a transcriptional increase in CD47 expression in LCC1 cells, implicating estrogen signaling in regulation of CD47 expression (Fig. 2b). Since LCC9 anti-estrogen-resistant cells were derived from LCC1 cells exposed to incrementally increased doses of Faslodex® (fulvestrant; ICI182.780), this finding supports our hypothesis that development of anti-estrogen resistance may involve increased CD47 expression. Knockdown of GRP78 by RNAi resulted in decreased CD47 expression in LCC9 cells and prevented tamoxifen-mediated induction of CD47 transcription in LCC1 cells (Fig. 2b & c). We confirmed knockdown of GRP78 protein levels in both cell lines (Fig. 2d & e).

In the paper by Cook et al., GRP78 knockdown sensitized resistant orthothopic breast tumors to tamoxifen therapy [3]. Taken together, these data suggest that sensitization of breast tumors to tamoxifen that is mediated by GRP78 knockdown may be due, in part, to CD47 regulation. LCC9 cells have more CD47 localized on the cell surface compared to LCC1 cells (Fig. 2f & g). Moreover, GRP78 knockdown reduced the amount of CD47 on the cell surface in LCC1 and LCC9 cell lines (Fig. 2h & i) versus controls (transfected breast cancer cells).

CD47 levels in response to anti-estrogen therapy may also be regulated by expression of CD47’s ligand, thrombopospondin-1 (TSP1). Inhibition of GRP78 or the combination of tamoxifen and GRP78 knockdown resulted in a 2-fold increase in tissue expression of TSP1, indicating that increased macrophage infiltration and enhanced therapeutic sensitization mediated by GRP78 knockdown may be due to increased TSP1 in tumor tissue (Fig. 2j). Elevated levels of TSP1 are associated with reduced growth of breast and other cancer types, via inhibition of angiogenesis and other pro-inflammatory mechanisms [17, 18]. Previous studies indicate anti-estrogen therapy induced TSP1 expression in several breast cancer cell lines [19, 20], however these studies were performed in endocrine therapy-sensitive cell lines. The decrease in TSP1 protein levels we observed in tamoxifen-treated resistant orthotopic breast tumors may be a potential molecular mechanism to maintain the endocrine therapy-resistant phenotype. Martin-Manso et al. showed that elevated expression of TSP1 in breast tumors led to increased M1 macrophage infiltration, and exogenous addition of TSP1 increased the cytolytic capacity of macrophages against breast carcinoma cell lines [21]. These results suggest that TSP1 may regulate CD47 levels to improve macrophage-mediated cancer cell killing beyond the well-described SIRPα-CD47 interaction, providing a new mechanism to explain the role of CD47 in innate anti-tumor responses.

In the paper by Cook et al., expression of CD47, calreticulin, and CD68 were measured in normal mammary gland tissues after targeting GRP78 through antisense oligomers [3]. Elevated infiltration of CD68+ macrophages was observed after GRP78 targeting. However, targeting GRP78 increased CD47 and calreticulin levels in normal mammary gland tissue, unlike tumor tissue. These data suggest that while macrophage infiltration is increased in both normal and malignant mammary gland tissue, the normal mammary gland is protected from deleterious effects through elevated CD47 protein expression [3]. The differential regulation of CD47 expression by GRP78 also may explain why CD47 blockade in preclinical models resulted in tumor sensitization to ionizing radiation, but protected soft tissues and bone marrow from toxicity [5]. Resistance to tamoxifen therapy is mediated in part by drug-induced pro-survival autophagy [22]. Moreover, knockdown of GRP78 inhibited drug-induced autophagic signaling, resulting in restoration of therapeutic sensitivity [2]. We also demonstrated that the radioprotection of normal tissue by CD47 targeting is mediated by upregulation of autophagy [23, 24]. Autophagy is tightly linked to the regulation of immunogenic cell death a previous study found that that ROS-stimulated ER stress induction of immunogenic cell death was associated with a decrease in CD47 expression indicating a possible mechanism for the differential regulation of cell survival [25]. The complete mechanism of dual effects on cell survival behind CD47 blockade is not completely understood, but CD47 may differentially regulate autophagy in normal and tumor tissue through cross-talk with the UPR pathway.

There are over 246,000 new cases of breast cancer diagnosed annually in the United States [26]. Advanced breast cancer largely remains an incurable disease. Since about 70% of all breast cancer cases are ER+, development of anti-estrogen resistance is a major factor driving breast cancer mortality. Therefore, new biomarkers are needed to identify drug-resistant tumors as well as novel therapeutic strategies. We have now identified co-expression of GRP78 and CD47 as a novel biomarker predicting decreased overall survival and reduced therapeutic responsiveness in ER+ breast cancer. Breast cancer is not known to be an immunogenic tumor type; however, Cook et al. showed that targeting GRP78 and the UPR pathway results in elevated macrophage recruitment [3]. Our data presented here shows that this result may be mediated in part by regulation of TSP1/CD47 signaling. Most of the literature describes CD47 as a marker of “self” that promotes tumor escape from macrophage immunosurveillance. The data presented by Cook et al. show that CD47 is regulated by lipid metabolism and UPR signaling, suggesting a new paradigm that may elucidate novel pathways to treat acquired resistance to anti-estrogen therapy, and thus improve clinical responses of breast cancer patients.

## Materials & methods

### Cancer patient survival database

To determine effects of co-expression of CD47 and GRP78 on relapse-free survival in cohorts of breast cancer, we used the KM plotter database (www.kmplot.com) [10]. The prognostic value of gene expression was assessed using the JetSet best probe set, which uses scoring methods to assess each probe set for specificity, coverage, and degradation resistance allowing selection of the optimal probe set for each gene [11, 27]. The cutoff method of the KM plot data base uses the median (or upper/lower quartile) sample for dividing the samples into high- and low-expression groups [10]. The IDs of Affymetrix probe analysis used were 21936_at (GRP78,HSPA5, BiP) 230031_at (HSPA5), and 226016_at (CD47). To confirm KM plot data we used the www.cbioportal.org database which provides visualization, analysis and download of large-scale cancer genomics data sets [13, 14]. The overall survival (OS) effect of co-expression of CD47 and GRP78 was assessed by selecting the Molecular Taxonomy of Breast Cancer International Consortium (METABRIC) [28, 29] with z-score threshold ±2.0 fold mRNA expression.

### Gene and Protein expression

Staining of tissue sections was performed as previously described [30]. Briefly, slides were deparaffinized in xylene and rehydrated in graded alcohol. Antigen retrieval was performed using microwave antigen retrieval method with Target Retrieval Solution, pH 6.10. Endogenous peroxidase activity was quenched by 0.3% H_2_O_2_ in water. After washing the slides to reduce non-specific binding, they were incubated with specific antibodies to thrombospondin-1 (Clone A6.1 Santa Cruz Biotechnology). Staining was done using DAB as the chromogen. mRNA expression of CD47 was assessed by real-time PCR using specific primers (Forward Primer 5′-AGCATGGAATGACGACAGTG-3′, reverse primer 5′-GATGTGGCCCCTGGTAGC-3′). Cell surface expression of CD47 was measured using a BD LSRFortessa X-20 Analyzer Flow Cytometer, staining of CD47 was performed using FITC Human Antibody B6H12 (Biolegend). Confirmation of GRP78 knockdown was performed by Western blot hybridization using a GRP78 antibody from Cell Signaling Technologies.

## Abbreviations

ER+: Estrogen receptor alpha positive
GRP: Glucose regulated protein
RFS: Relapse-free survival
TSP1: Thrombospondin-1
UPR: Unfolded protein response
